# Supplementary material for: Assessing antigenic drift and phylogeny of influenza A (H1N1) pdm09 virus in Kenya using HA1 sub-unit of the hemagglutinin gene
Source: PLoS One. 2020 Feb 11;15(2):e0228029. doi: 10.1371/journal.pone.0228029 (PMC7012450; doi:10.1371/journal.pone.0228029)
Supplement: S1 Table — (DOCX) [file pone.0228029.s001.docx]

**S1 Table.** **Accession numbers in GISAID and GenBank databases of HA gene sequences of A/H1N1 pdm09 reference strains included in the analysis.**

CY121816 CY238222 CY264068 EPI1039619 EPI1056459 EPI1063021

EPI1078475 EPI1089683 EPI1105955 EPI1114306 EPI1136978 EPI1141060

EPI1145477 EPI1152395 EPI1152647 EPI1161910 EPI1168899 EPI1181373

EPI1203066 EPI1210929 EPI1212842 EPI1214383 EPI1227801 EPI1229653

EPI1230811 EPI1244270 EPI1250803 EPI1255578 EPI1258971 EPI1267197

EPI1267228 EPI1272016 EPI1277961 EPI1282115 EPI1310729 EPI1313915

EPI1313922 EPI1326095 EPI1326796 EPI1328173 EPI1328786 EPI1555520

EPI280341 EPI319447 EPI319590 EPI326206 EPI390473 EPI630610

EPI630684 EPI684339 EPI687821 EPI694825 EPI712028 EPI720442

EPI742220 EPI746515 EPI758495 EPI765066 EPI770131 EPI774694

EPI859057 EPI874346 EPI875266 EPI879205 EPI892786 EPI917635

GQ169382 KP941689 KU933485 KY117023
