# Supplementary material for: Assessing antigenic drift and phylogeny of influenza A (H1N1) pdm09 virus in Kenya using HA1 sub-unit of the hemagglutinin gene
Source: PLoS One. 2020 Feb 11;15(2):e0228029. doi: 10.1371/journal.pone.0228029 (PMC7012450; doi:10.1371/journal.pone.0228029)
Supplement: S1 Fig — (PDF) [file pone.0228029.s002.pdf]

S1 Fig. Alignment of HA1 amino acid sequences of A/H1N1 pdm09 strains isolated in Kenya in 2015 with foreign strains, relative to vaccine virus A/California/7/2009.

```
                                  10         20         30         40         50         60         70         80         90        100
                          ....|....| ....|....| ....|....| ....|....| ....|....| ....|....| ....|....| ....|....| ....|....| ....|....|
A_California_07_2009      DTLCIGYHAN NSTDTVDTVL EKNVTVTHSV NLLEDKHNGK LCKLRGVAPL HLGKCNIAGW ILGNPECESL STASSWSYIV ETPSSDNGTC YPGDFIDYEE
A_Kenya_001_2015          .......... .......... .......... .......... .......P.. .......... .......... .......... ..SN...... ......N...
A_Kenya_004_2015          .......... .......... .......... .......... .......... .......... .......... .......... ..SN...... ......N...
A_Kenya_006_2015          .......... .......... .......... .......... .......... .......... .......... .......... ..SN...... ......N...
A_Kenya_002_2015          .......... .......... .......... .......... ....K..... .......... .......... .......... ..SN...... ......N...
A_Kenya_005_2015          .......... .......... .......... .......... ....K..... .......... .......... .......... ..SN...... ......N...
A_Cameroon_15V-3038_2015  .......... .......... .......... .......... .......... .......... .......... .......... ..S....... ......N...
A_Ghana_DILI-15-1165_2015 .......... .......... .......... .......... .......... .......... .......... .......... ..SN...... ......N...
A_South_Africa_5287_2015  .......... .......... .......... .......... .......... .......... .......... .......... ..S....... ......N...
A_England_427_2015        .......... .......... .......... .......... .......... .......... .......... .......... ..SN...... ......N...
A_Singapore_KK967_2015    .......... .......... .......... .......... .......... .......... .......... .......... ..S....... ......N...
A_New_York_61_2015        .......... .......... .......... .......... .......... .......... .......... .......... ..SN...... ......N...
A_Brazil_0638_2015        .......... .......... .......... .......... .......... .......... .......... .......... ..SN...... ......N...
A_Brisbane_202_2015       .......... .......... .......... .......... .......... .......... .......... .......... ..SN...... ......N...
A_Michigan_45_2015        .......... .......... .......... .......... .......... .......... .......... .......... ..SN...... ......N...
A_N.Ireland_60160398_2015 .......... .......... .......... .......... .......... .......... .......... .......... ..SN...... ......N...
A_England_357_2015        .......... .......... .......... .......... .......... .......... .......... .......... ..S....... ......N...
A_South_Africa_R3723_2015 .......... .......... .......... .......... .......... .......... ........P. .......... ..S....... ......N...
A_Egypt_6283_2015         .......... .......... .......... .......... .......... .......... .......... .......... ..SN...... ......N...

                                 110        120        130        140        150        160        170        180        190        200
                          ....|....| ....|....| ....|....| ....|....| ....|....| ....|....| ....|....| ....|....| ....|....| ....|....|
A_California_07_2009      LREQLSSVSS FERFEIFPKT SSWPNHDSNK GVTAACPHAG AKSFYKNLIW LVKKGNSYPK LSKSYINDKG KEVLVLWGIH HPSTSADQQS LYQNADAYVF
A_Kenya_001_2015          .......... .......... ........D. .......... .......... .......... ..Q....... .......... ....T..... ..........
A_Kenya_004_2015          .......... .......... .......... .......... .......... .......... ..Q....... .......... ....T..... ..........
A_Kenya_006_2015          .......... .......... .......... .......... .......... .......... ..Q....... .......... ....T..... ..........
A_Kenya_002_2015          .......... .......... .......... ..P....... .......... .......... ..Q....... .......... ....T..... ..........
A_Kenya_005_2015          .......... .......... .......... .......... .......... .......... ..Q....... .......... ....T..... ..........
A_Cameroon_15V-3038_2015  .......... .......... .......... .......... .......... .......... ..Q....... .......... ....T..... ..........
A_Ghana_DILI-15-1165_2015 .......... .......... .......... .......... .......... .......... ..Q....... .......... ....T..... ..........
A_South_Africa_5287_2015  .......... .......... .......... .......... .......... .......... ..Q....... .......... ....T..... ..........
A_England_427_2015        .......... .......... .......... .......... .......... .......... .NQ....... .......... ....T..... ..........
A_Singapore_KK967_2015    .......... .......... ........D. .......... .......... .......... ..Q....... .......... ....T...R. ..........
A_New_York_61_2015        .......... .......... .......... .......... .......... .......... .NQ....... .......... ....T..... ..........
A_Brazil_0638_2015        .......... .......... .......... .......... .......... .......... ..Q....... .......... ....T..... ..........
A_Brisbane_202_2015       .......... .......... .......... .......... .......... .......... .NQ....... .......... ....T..... ..........
A_Michigan_45_2015        .......... .......... .......... .......... .......... .......... .NQ....... .......... ....T..... ..........
A_N.Ireland_60160398_2015 .......... .......... .......... .......... .......... .......... .NQ....... .......... ....T..... ..........
A_England_357_2015        .......... .......... .......... .......... ..G....... .T........ ..Q....... ..I....... ....T..... ..........
A_South_Africa_R3723_2015 .......... .........A .......... .......... .......... .......... ..Q....... .......... ....T..... ..........
A_Egypt_6283_2015         .......... .......... .......... .......... .......... .......... .NQ....... .......... ....T..... ..........

                                 210        220        230        240        250        260        270        280        290        300
                          ....|....| ....|....| ....|....| ....|....| ....|....| ....|....| ....|....| ....|....| ....|....| ....|....|
A_California_07_2009      VGSSRYSKKF KPEIAIRPKV RDQEGRMNYY WTLVEPGDKI TFEATGNLVV PRYAFAMERN AGSGIIISDT PVHDCNTTCQ TPKGAINTSL PFQNIHPITI
A_Kenya_001_2015          ..T....... .......... .......... .......... .......... .....T.... .......... .......... ..E....... ..........
A_Kenya_004_2015          ..T....... .......... .......... .......... .......... .....T.... .......... .......... ..E....... ..........
A_Kenya_006_2015          ..T....... .......... .......... .......... .......... .....T.... .......... .......... ..E....... ..........
A_Kenya_002_2015          ..T....... .......... .......... .......... .......... .....T.... .......... .......... ..E....... ..........
A_Kenya_005_2015          ..T....... .......... .......... .......... .......... .....T.... .......... .......... ..E....... ..........
A_Cameroon_15V-3038_2015  ..T....... .......... .......... .......... .......... .....T.... .......... .......... ..E....... ..........
A_Ghana_DILI-15-1165_2015 ..T....... .......... .......... .......... .......... .....T.... .......... .......... ..E....... ..........
A_South_Africa_5287_2015  ..T....... .......... .......... .......... .......... .....T.... .......... .......... ..E....... ..........
A_England_427_2015        ..T....... .....T.... .G........ .......... .......... .....T.... .......... .......... ..E....... ..........
A_Singapore_KK967_2015    ..T....... .......... .......... .......... .......... .....T.... .......... .......... ..E....... ..........
A_New_York_61_2015        ..T....... .....T.... .......... .......... .......... .....T.... .......... .......... ..E....... ..........
A_Brazil_0638_2015        ..T....... .......... .......... .......... .......... .....T.... .......... .......... ..E....... ..........
A_Brisbane_202_2015       ..T....... .....T.... .......... .......... .......... .....T.... .......... .......... ..E....... ..........
A_Michigan_45_2015        ..T....... .....T.... .......... .......... .......... .....T.... .......... .......... ..E....... ..........
A_N.Ireland_60160398_2015 ..T....... .....T.... .......... .......... .......... .....T.... .......... .......... ..E....... ..........
A_England_357_2015        ..T....... .......... .......... .......... .......... .....T.... .......... .......... ..E....... ..........
A_South_Africa_R3723_2015 ..T....R.. .......... .......... ....D..... .......... .....T.... .......... .......... ..E....... ..........
A_Egypt_6283_2015         ..T....... .....T.... .......... .......... .......... .....T.... .......... .......... .SE....... ..........

                                 310        320
                          ....|....| ....|....| ....|..
A_California_07_2009      GKCPKYVKST KLRLATGLRN IPSIQSR
A_Kenya_001_2015          .......... .......... V......
A_Kenya_004_2015          .......... .......... V......
A_Kenya_006_2015          .......... .......... V......
A_Kenya_002_2015          .......... .......... V.....K
A_Kenya_005_2015          .......... .......... V......
A_Cameroon_15V-3038_2015  .......... .......... V......
A_Ghana_DILI-15-1165_2015 .......... .......... V......
A_South_Africa_5287_2015  .......... .......... V......
A_England_427_2015        .......... .......... V......
A_Singapore_KK967_2015    .......... .......... V......
A_New_York_61_2015        .......... .......... V......
A_Brazil_0638_2015        .......... .......... V......
A_Brisbane_202_2015       .......... .......... V......
A_Michigan_45_2015        .......... .......... V......
A_N.Ireland_60160398_2015 .......... .......... V......
A_England_357_2015        .......... .......... V......
A_South_Africa_R3723_2015 .......... .......... V......
A_Egypt_6283_2015         .......... .......... V......
```
