# Supplementary material for: Assessing antigenic drift and phylogeny of influenza A (H1N1) pdm09 virus in Kenya using HA1 sub-unit of the hemagglutinin gene
Source: PLoS One. 2020 Feb 11;15(2):e0228029. doi: 10.1371/journal.pone.0228029 (PMC7012450; doi:10.1371/journal.pone.0228029)
Supplement: S2 Fig — (PDF) [file pone.0228029.s003.pdf]

S2 Fig. Alignment of HA1 amino acid sequences of A/H1N1 pdm09 strains isolated in Kenya in 2016 with foreign strains, relative to vaccine virus A/California/7/2009.

```
                                    10         20         30         40         50         60         70         80         90        100
                           ....|....| ....|....| ....|....| ....|....| ....|....| ....|....| ....|....| ....|....| ....|....| ....|....|
A_California_07_2009       DTLCIGYHAN NSTDTVDTVL EKNVTVTHSV NLLEDKHNGK LCKLRGVAPL HLGKCNIAGW ILGNPECESL STASSWSYIV ETPSSDNGTC YPGDFIDYEE
A_Kenya_022_2016           .......... .......... .......... .......... .......... .......... .........F .......... ..SN...... ......N...
A_Kenya_017_2016           .......... .......... .......... .......... .......... .......... .......... .......... ..SN...... ......N...
A_Ghana_FS-16-0320_2016    .......... .......... .......... .......... .......... .......... .......... .......... ..SN...... ......N...
A_South-Africa_R5236_2016  .......... .......... .......... .......... .......... .......... .......... .......... ..SN...... ......N...
A_England_676_2016         .......... .......... .......... .......... .......... .......... .......... .......... ..SN...... ......N...
A_Phuket_320_2016          .......... .......... .......... .....E.... .......... .......... .......... .......... ..SN...... ......N...
A_Maryland_28_2016         .......... .......... .......... .......... .......... .......... .......... .......... ..SN...... ......N...
A_Sydney_344_2016          .......... .......... .......... .......... .......... .......... .......... .......... ..SN...... ......N...
A_Singapore_KK0974_2016    .......... .......... .......... .......... .......... .......... .......... .......... ..SN...... ......N...
A_Macao_611368_2016        .......... .......... .......... .......... .......... .......... .......... .......... ..S....... ......N...
A_Moscow_1_2016            .......... .......... .......... .......... .......... .......... .......... .......... ..SN...... ......N...
A_Brazil_1517_2016         .......... .......... .......... .......... .......... .......... .......... .......... ..SN...... ......N...

                                   110        120        130        140        150        160        170        180        190        200
                           ....|....| ....|....| ....|....| ....|....| ....|....| ....|....| ....|....| ....|....| ....|....| ....|....|
A_California_07_2009       LREQLSSVSS FERFEIFPKT SSWPNHDSNK GVTAACPHAG AKSFYKNLIW LVKKGNSYPK LSKSYINDKG KEVLVLWGIH HPSTSADQQS LYQNADAYVF
A_Kenya_022_2016           .......... .......... .......... .......... .......... .......... .RQ....... .......... ....T..... ..........
A_Kenya_017_2016           .......... .......... .......... .......... .......... .....K.... ..Q....... .......... ....T..... ..........
A_Ghana_FS-16-0320_2016    .......... .......... .......... .......... .......... .......... .NQ....... .......... ....T..... ..........
A_South-Africa_R5236_2016  .......... .......... .......... .......... .......... .......... .NQ....... .......... ....T..... ..........
A_England_676_2016         .......... .......... .......... .......... .......... .......... .NQ....... .......... ....T..... ..........
A_Phuket_320_2016          .......... .......... .......... .......... .......... .......... .NQ....... .......... ....T..... ..........
A_Maryland_28_2016         .......... .......... .......... .......... .......... .......... .NQ..V.... .......... ..P.T..... ..........
A_Sydney_344_2016          .......... .......... .......... .......... .......... .......... .NQ....... .......... ....T..... ..........
A_Singapore_KK0974_2016    .......... .......... .......... .......... .......... .......... .NQ....... .......... ..A.T..... ..........
A_Macao_611368_2016        .......... .......... .......... .......... .......... .T........ ..Q....... ..I....... ....T..... ..........
A_Moscow_1_2016            .......... .......... .......... .......... .......... .......... .NQ....... .......... ....T..... ..........
A_Brazil_1517_2016         .......... .......... .......... .......... .......... .......... .NQ....... .......... ....T..... ..........

                                   210        220        230        240        250        260        270        280        290        300
                           ....|....| ....|....| ....|....| ....|....| ....|....| ....|....| ....|....| ....|....| ....|....| ....|....|
A_California_07_2009       VGSSRYSKKF KPEIAIRPKV RDQEGRMNYY WTLVEPGDKI TFEATGNLVV PRYAFAMERN AGSGIIISDT PVHDCNTTCQ TPKGAINTSL PFQNIHPITI
A_Kenya_022_2016           ..T....... .......... .......... .......... .......... .....T.... .......... .......... ..E....... ..........
A_Kenya_017_2016           ..T....... .......... .......... .......... .......... .....TL... .......... .......... ..E....... ..........
A_Ghana_FS-16-0320_2016    ..T....... .....T.... .......... .......... .......... .....T.... .......... .......... ..E....... ..........
A_South-Africa_R5236_2016  ..T....... .....T.... .......... .......... .......... .....T.... .......... .......... ..E....... ..........
A_England_676_2016         ..T....... .....T.... .......... .......... .......... .....T.... .......... .......... ..E....... ..........
A_Phuket_320_2016          ..T....... ....GT.... .......... .I........ .......... .....T.... .......... .......... ..E....... ..........
A_Maryland_28_2016         ..T.K..... .....T.... .......... .......... .......... .....T.... .......... .......... ..E....... ..........
A_Sydney_344_2016          ..T....... .....T.... .......... .......... .......... .....T.... .......... .......... ..E....... ..........
A_Singapore_KK0974_2016    ..T....... .....T.... .......... .......... .......... .....T.... ........N. .......... ..E....... ..........
A_Macao_611368_2016        ..T....... .......... .......... .......... .......... .....T.... S......... .......... ..E....... ..........
A_Moscow_1_2016            ..T....... .....T.... .......... .......... .......... .....TI... .......... .......... ..E....... ..........
A_Brazil_1517_2016         ..T....... .....T.... .......... .......... .......... .....T.... .......... .......... ..D....... ..........

                                   310        320
                           ....|....| ....|....| ....|..
A_California_07_2009       GKCPKYVKST KLRLATGLRN IPSIQSR
A_Kenya_022_2016           .......... .......... V......
A_Kenya_017_2016           .......... .......... V......
A_Ghana_FS-16-0320_2016    .......... .......... V......
A_South-Africa_R5236_2016  .......... .......... V......
A_England_676_2016         .......... .......... V......
A_Phuket_320_2016          .......... .......... V......
A_Maryland_28_2016         .......... .......... V......
A_Sydney_344_2016          .......... .......... V......
A_Singapore_KK0974_2016    .......... .......... V......
A_Macao_611368_2016        .......... .......... V......
A_Moscow_1_2016            .......... .......... V......
A_Brazil_1517_2016         .......... .......... V......
```
