# Supplementary material for: Assessing antigenic drift and phylogeny of influenza A (H1N1) pdm09 virus in Kenya using HA1 sub-unit of the hemagglutinin gene
Source: PLoS One. 2020 Feb 11;15(2):e0228029. doi: 10.1371/journal.pone.0228029 (PMC7012450; doi:10.1371/journal.pone.0228029)
Supplement: S3 Fig — (PDF) [file pone.0228029.s004.pdf]

S3 Fig. Alignment of HA1 amino acid sequences of A/H1N1 pdm09 strains isolated in Kenya in 2017 with foreign strains, relative to vaccine virus A/Michigan/45/2015.

```
                                    10         20         30         40         50         60         70         80         90        100
                            ....|....| ....|....| ....|....| ....|....| ....|....| ....|....| ....|....| ....|....| ....|....| ....|....|
A_Michigan_45_2015_6B.1     DTLCIGYHAN NSTDTVDTVL EKNVTVTHSV NLLEDKHNGK LCKLRGVAPL HLGKCNIAGW ILGNPECESL STASSWSYIV ETSNSDNGTC YPGDFINYEE
A_Kenya_045_2017            .......... .......... .......... .......... .......... .......... .......... ...R...... .......... ..........
A_Kenya_057_2017            .......... .......... .......... .......... .......... .......... .......... ...R...... .......... ..........
A_Cameroon_9535_2017        .......... .......... .......... .......... .......... .......... .......... ...R...... .......... ..........
A_Cameroon_9887_2017        .......... .......... .......... .......... .......... .......... .......... ...R...... .......... ..........
A_Ghana_3688_2017           .......... .......... .......... .......... .......... .......... .......... .......... .......... ..........
A_England_199_2017          .......... .......... .......... .......... .......... .......... .......... ...R...... .......... ..........
A_Singapore_NUH0026_2017    .......... .......... .......... .......... .......... .......... .......... ...R...... .......... ..........
A_Perth_346_2017            .......... .......... .......... .......... ....K..... .......... .......... ...R...... .......... ..........
A_Michigan_272_2017_6B.1    .......... .......... .......... .......... .......... .......... .......... ...R...... .......... ..........
A_Hawaii_25_2017_6B.1       .......... .......... .......... .......... .......... .......... .......... .......... .......... ..........
A_Michigan_284_2017         .......... .......... .......... .......... .......... .......... .......... ...R...... .......... ..........
A_Maryland_94_2017          .......... .......... .......... .......... .......... .......... .......... ...R...... .......... ..........
A_South_Africa_R07198_2017  .......... .......... .......... .......... .......... .......... .......... ...R...... .......... ..........
A_Egypt_799_2017            .......... .......... .......... .......... .......... .......... .......... ...R...... .......... ..........
A_England_196_2017          .......... .......... .......... .......... .......... .......... .......... ...R...... .......... ..........
A_Sydney_550_2017           .......... .......... .......... .......... .......... .......... .......... ...R...... .......... ..........
A_Brazil_5026_2017          .......... .......... .......... .......... .......... .......... .......... ...R...... .......... ..........
A_Hawaii_54_2017            .......... .......... .......... .......... .......... .......... .......... ...R...... .......... ..........
A_Tunisia_12248_2017        .......... .......... .......... .......... ......I... .......... .......... ...R...... .......... ..........
A_India_P1727283_2017       .......... .......... .......... .......... .......... .......... .......... ...R...... .......... ..........
A_Alaska_100_2017           .......... .......... .......... .......... .......... .......... .......... ...R...... .......... ..........

                                   110        120        130        140        150        160        170        180        190        200
                            ....|....| ....|....| ....|....| ....|....| ....|....| ....|....| ....|....| ....|....| ....|....| ....|....|
A_Michigan_45_2015_6B.1     LREQLSSVSS FERFEIFPKT SSWPNHDSNK GVTAACPHAG AKSFYKNLIW LVKKGNSYPK LNQSYINDKG KEVLVLWGIH HPSTTADQQS LYQNADAYVF
A_Kenya_045_2017            .......... .......... .......... .......... .......... .......... ...T...... .......... .......... ..........
A_Kenya_057_2017            .......... .......... .......... .......... .......... .......... ...T...... .......... .......... ..........
A_Cameroon_9535_2017        .......... .......... .......... .......... .......... .......... .......... .......... .....T.... ..........
A_Cameroon_9887_2017        .......... .......... .......... .......... .......... .......... .......... .......... .....T.... ..........
A_Ghana_3688_2017           .......... .......... .......... .......... .......... .......... .......... .......... .......... ..........
A_England_199_2017          .......... .......... .......... .......... .......... .......... ...T...... .......... .......... ..........
A_Singapore_NUH0026_2017    .......... .......... .......... .......... .......... .......... .......... .......... .......... ..........
A_Perth_346_2017            .......... .......... .......... .......... .......... .......... ...T...... .......... .......... ..........
A_Michigan_272_2017_6B.1    .......... .......... .......... .......... .......... .......... ...T...... .......... .......... ..........
A_Hawaii_25_2017_6B.1       .......... .......... .......... .......... .......... .......... .....V.... .......... ..P....... ..........
A_Michigan_284_2017         .......... .......... .......... .......... .......... .......... ...T...G.. .......... .......... ..........
A_Maryland_94_2017          .......... .......... .......... .......... .......... .......... ...T...... .......... .......... ..........
A_South_Africa_R07198_2017  .......... .......... .......... .......... .......... .......... ...T...... .......... .......... ..........
A_Egypt_799_2017            .......... .......... .......... .......... .......... .......... ...T...... .......... .......... ..........
A_England_196_2017          .......... .......... .......... .......... .......... .......... ...T...... .......... .......... ..........
A_Sydney_550_2017           .......... .......... .......... .......... .......... .......... ...T...... .......... .......... ..........
A_Brazil_5026_2017          .......... .......... .......... .......... .......... .......... ...T...... .......... .......... ..........
A_Hawaii_54_2017            .......... .......... .......... .......... .......... .......... ...T...... .......... .......... ..........
A_Tunisia_12248_2017        .......... .......... .......... .......... .......... .......... ...T...... .......... .......... ..........
A_India_P1727283_2017       .......... .......... .......... .......... .......... .......... ...T...... .......... .......... ..........
A_Alaska_100_2017           .......... ........A. .......... ......S... E......... .......... ...T...... .......... .......... ..........

                                   210        220        230        240        250        260        270        280        290        300
                            ....|....| ....|....| ....|....| ....|....| ....|....| ....|....| ....|....| ....|....| ....|....| ....|....|
A_Michigan_45_2015_6B.1     VGTSRYSKKF KPEIATRPKV RDQEGRMNYY WTLVEPGDKI TFEATGNLVV PRYAFTMERN AGSGIIISDT PVHDCNTTCQ TPEGAINTSL PFQNIHPITI
A_Kenya_045_2017            .......... .......... .......... .......... ......K... .......... .......... .......... .......... ....V.....
A_Kenya_057_2017            .......... .......... .......... .......... .......... .......... .......... .......... .......... ....V.....
A_Cameroon_9535_2017        .......... X......... .......... .......... .......... .......... .......... .......... .......... ....V.....
A_Cameroon_9887_2017        .......... .......... .......... .......... .......... .......... .......... .......... .......... ....V.....
A_Ghana_3688_2017           .......... .......... .......... .......... .......... .......... .......... .A........ .......... ..........
A_England_199_2017          .......... .......... .......... .......... .......... .......... .......... .......... .......... ....V.....
A_Singapore_NUH0026_2017    .......... .......... .......... .......... .......... .......... .......... ..Q....... .......... ....V.....
A_Perth_346_2017            .......... .......... .......... .......... .......... .......... .......... .......... .......... ....V.....
A_Michigan_272_2017_6B.1    .......... .......... .......... .......... .......... .......... .......... .......... .......... ....V.....
A_Hawaii_25_2017_6B.1       ....K..... .......... .......... .......... .......... .......... .......... .......... .......... ..........
A_Michigan_284_2017         .......... .......... .......... .......... .......... .......... .......... .......... .......... ....V.....
A_Maryland_94_2017          .......... .......... .......... .......... .......... .......... .......... .......... .....V.... ....V.....
A_South_Africa_R07198_2017  .......... .......... .......... .......... .......... .......... .......... .......... .......... ....V.....
A_Egypt_799_2017            .......... .......... .......... .......... .......... .......... .......... .......... .......... ....V.....
A_England_196_2017          .......... .......... .......... .......... .......... .......... .......... .......... .......... ....V.....
A_Sydney_550_2017           .......... .......... .......... .......... .......... .......... .......... .......... .......... ....V.....
A_Brazil_5026_2017          .......... .......... .......... .......... .......... .......... .......... .......... .......... ....V.....
A_Hawaii_54_2017            .......... .......... .......... .......... .......... .......... .......... .......... .......... ....V.....
A_Tunisia_12248_2017        .......... .......... .......... .......... .......... .......... .......... .......... .......... ....V.....
A_India_P1727283_2017       .......... .......... .......... .......... .......... .......... .......... .......... .......... ....V.....
A_Alaska_100_2017           .......... .......... .......... .......... .......... .......... .......... ..Y....... .......... ....V.....
```

```
                                   310        320
                            ....|....| ....|....| ....|..
A_Michigan_45_2015_6B.1     GKCPKYVKST KLRLATGLRN VPSIQSR
A_Kenya_045_2017            .......... .......... .......
A_Kenya_057_2017            .......... .......... .......
A_Cameroon_9535_2017        .......... .......... .......
A_Cameroon_9887_2017        .......... .......... .......
A_Ghana_3688_2017           .......... .......... ...V...
A_England_199_2017          .......... .......... .......
A_Singapore_NUH0026_2017    .......... .......... .......
A_Perth_346_2017            .......... .......... .......
A_Michigan_272_2017_6B.1    .......... .......... .......
A_Hawaii_25_2017_6B.1       .......... .......... .......
A_Michigan_284_2017         .......... .......... .......
A_Maryland_94_2017          .......... .......... .......
A_South_Africa_R07198_2017  .......... .......... .......
A_Egypt_799_2017            .......... .......... .......
A_England_196_2017          .......... .......... .......
A_Sydney_550_2017           .......... N......... .......
A_Brazil_5026_2017          .......... .......... .......
A_Hawaii_54_2017            .......... .......... .......
A_Tunisia_12248_2017        .......... .......... .......
A_India_P1727283_2017       .......... .......... .......
A_Alaska_100_2017           .......... .......... .......
```
