# Supplementary material for: Assessing antigenic drift and phylogeny of influenza A (H1N1) pdm09 virus in Kenya using HA1 sub-unit of the hemagglutinin gene
Source: PLoS One. 2020 Feb 11;15(2):e0228029. doi: 10.1371/journal.pone.0228029 (PMC7012450; doi:10.1371/journal.pone.0228029)
Supplement: S4 Fig — (PDF) [file pone.0228029.s005.pdf]

S4 Fig. Alignment of HA1 amino acid sequences of A/H1N1 pdm09 strains isolated in Kenya in 2018 with foreign strains, relative to vaccine virus A/Michigan/45/2015.

```
                                     10         20         30         40         50         60         70         80         90        100
                             ....|....| ....|....| ....|....| ....|....| ....|....| ....|....| ....|....| ....|....| ....|....| ....|....|
A_Michigan_45_2015_6B.1      DTLCIGYHAN NSTDTVDTVL EKNVTVTHSV NLLEDKHNGK LCKLRGVAPL HLGKCNIAGW ILGNPECESL STASSWSYIV ETSNSDNGTC YPGDFINYEE
A_Kenya_066_2018             .K........ .......... .......... .......... .......... .......... .......... ...R...... .......... ..........
A_Kenya_047_2018             .......... .......... .......... .......... .......... .......... .......... ...R...... .......... ..........
A_Kenya_059_2018             .......... .......... .......... .......... .......... .......... .......... ...R...... .......... ..........
A_Kenya_045_2018             .......... .......... .......... .......... .......... .......... .......... ...R...... .......... ..........
A_Kenya_046_2018             .......... .......... .......... .......... .......... .......... .......... ...R...... .......... ..........
A_Kenya_039_2018             .......... .......... .......... .......... .......... .......... .......... ...R...... .......... ..........
A_Kenya_041_2018             .......... .......... .......... .......... .......... .......... .......... ...R...... .......... ..........
A_Kenya_027_2018             .......... .......... .......... .....R.... .......... .......... .......... ...R...... .......... ..........
A_Kenya_055_2018             .K........ .......... .......... .......... .......... .......... .......... ...R...... .......... ..........
A_Kenya_060_2018             .K........ .......... .......... .......... .......... .......... .......... ...R...... .......... ..........
A_Kenya_056_2018             .K........ .......... .......... .......... .......... .......... .......... ...R...... .......... ..........
A_Kenya_063_2018             .K........ .......... .......... .......... ....K..... .......... .......... ...R...... .......... ..........
A_Kenya_028_2018             .K........ .......... .......... .......... .......... .......... .......... ...R...... .......... ..........
A_Kenya_013_2018             .K........ .......... .......... .......... .......... .......... .......... ...R...... .......... ..........
A_Kenya_065_2018             .K........ .......... .......... .......... .......... .......... .......... ...R...... .......... ..........
A_Kenya_064_2018             .K........ .......... .......... .......... .......... .......... .......... ...R...... .......... ..........
A_Kenya_062_2018             .K........ .......... .......... .......... .......... .......... .......... ...R...... .......... ..........
A_Kenya_057_2018             .K........ .......... .......... .......... .......... .......... .......... ...R...... .......... ..........
A_Kenya_051_2018             .K........ .......... .......... .......... .......... .......... .......... ...R...... .......... ..........
A_Kenya_043_2018             .......... .......... .......... .......... .......... .......... .......... ...R...... .......... ..........
A_Kenya_042_2018             .......... .......... .......... .......... .......... .......... .......... ...R...... .......... ..........
A_Kenya_035_2018             .......... .......... .......... .......... .......... .......... .......... ...R...... .......... ..........
A_Kenya_036_2018             .......... .......... .......... .......... .......... .......... .......... ...R...... .......... ..........
A_Kenya_029_2018             .......... .......... .......... .......... .......... .......... .......... ...R...... .......... ..........
A_Kenya_025_2018             .......... .......... .......... .......... .......... .......... .......... ...R...... .......... ..........
A_Kenya_024_2018             .......... .......... .......... .......... .......... .......... .......... ...R...... .......... ..........
A_Kenya_020_2018             .......... .......... .......... .......... .......... .......... .......... ...R...... .......... ..........
A_Kenya_019_2018             .......... .......... .......... .......... .......... .......... .......... ...R...... .......... ..........
A_Kenya_017_2018             .......... .......... .......... .......... .......... .......... .......... ...R...... .......... ..........
A_Madagascar_883_2018        .......... .......... .......... .......... .......... .......... .......... ...R...... .......... ..........
A_South_Africa_5719_2018     .......... .......... .......... .......... ....G..... .......... .......... ..TR...... .......... ..........
A_South_Africa_R07331_2018   .......... .......... .......... .......... .......... .......... .......... ...R...... .......... ..........
A_Uganda_6825_2018           .......... .......... .......... .......... .......... .......... .......... ...R...... .......... ..........
A_Bangkok_99_2018            .......... .......... .......... .......... .......... .......... .......... ...R...... .......... ..........
A_New_York_50_2018           .......... .......... .......... .......... .......... .......... .......... ...R...... .......... ..........
A_Brazil_0382_2018           .......... .......... .......... .......... ....G..... .......... .......... ...R...... .......... ..........
A_Sydney_1034_2018           .......... .......... .......... .......... .......... .......... .......... ...R...... .......... ..........
A_Pennsylvania_01_2018       .......... .......... .......... .......... .......... .......... .......... ...R...... .......... ..........
A_Florida_69_2018            .......... .......... .......... .......... .......... .......... .......... ...R...... .......... ..........
A_Uganda_6100_2018           .......... .......... .......... .......... .......... .......... .......... ...R...... .......... ..........
A_Egypt_458_2018             .......... .......... .......... .......... .......... .......... .......... ...R...... .......... ..........
A_Zhejiang-CHEN_2018         .......... .......... .......... .......... .......... .......... .......... ...R...... .......... ..........
A_Moscow_96_2018             .......... .......... .......... .......... .......... .......... .......... ...R...... .......... ..........
A_Brazil_2299_2018           .......... .......... .......... .......... .......... .......... .......... ...R...... .......... ..........
A_Indiana_21_2018            .......... .......... .......... .......... .......... .......... .......... ...R...... .......... ..........
A_Wyoming_04_2018            .......... .......... .......... .......... .......... .......... .......... ...R...... .......... ..........
A_Ohio_30_2018               .......... .......... .......... .......... .......T.. .......... .......... ...R...... .......... ..........
A_New Jersey_19_2018         .......... .......... .......... .......... .......... .......... .......... ...R...... .......... ..........
A_Washington_73_2018         .......... .......... .......... .......... .......... .......... .......... ...R...... .......... ..........

                                    110        120        130        140        150        160        170        180        190        200
                             ....|....| ....|....| ....|....| ....|....| ....|....| ....|....| ....|....| ....|....| ....|....| ....|....|
A_Michigan_45_2015_6B.1      LREQLSSVSS FERFEIFPKT SSWPNHDSNK GVTAACPHAG AKSFYKNLIW LVKKGNSYPK LNQSYINDKG KEVLVLWGIH HPSTTADQQS LYQNADAYVF
A_Kenya_066_2018             .......... .........A .......... .......... .......... .......... ...T...... .......... .......... ..........
A_Kenya_047_2018             .......... .........A .......... ......S... E......... .......... ...T...... .......... .......... ..........
A_Kenya_059_2018             .......... .........A .......... ......S... E......... .......... ...T...... .......... .......... ..........
A_Kenya_045_2018             .......... .........A .......... ......S... E......... .......... ...T...... .......... .......... ..........
A_Kenya_046_2018             .......... .........A .......... ......S... E.......L. .......... ...T...... .......... .......... ..........
A_Kenya_039_2018             .......... .........A .......... ......S... E.......L. .......... ...T...... .......... .......... ..........
A_Kenya_041_2018             .......... .........A .......... ......S... E.......L. .......... ...T...... .......... .......... ..........
A_Kenya_027_2018             .......M.. .........A .......L.. .......... .......... .......... ...T...... .......... .......... ..........
A_Kenya_055_2018             .......... .........A .......... .......... .......... .......... ...T...... .......... .......... ..........
A_Kenya_060_2018             .......... .........A .......... .......... .......... .......... ...T...... .......... .......... ..........
A_Kenya_056_2018             .......... .........A .......... .......... .......... .......... ...T...... .......... .......... ..........
A_Kenya_063_2018             .......... .........A .......... .......... .......... .......... ...T...... .......... .......... ..........
A_Kenya_028_2018             .......... .........A .......... .......... .......... .......... ...T...... .......... .......... ..........
A_Kenya_013_2018             .......... .........A .......... .......... .......... .......... ...T...... .......... .......... ..........
A_Kenya_065_2018             .......... .........A .......... .......... .......... .......... ...T...... .......... .......... ..........
A_Kenya_064_2018             .......... .........A .......... .......... .......... .......... ...T...... .......... .......... ..........
A_Kenya_062_2018             .......... .........A .......... .......... .......... .......... ...T...... .......... .......... ..........
A_Kenya_057_2018             .......... .........A .......... .......... .......... .......... ...T...... .......... .......... ..........
A_Kenya_051_2018             .......... .........A .......... .......... .......... .......... ...T...... .......... .......... ..........
A_Kenya_043_2018             .......... .......... .......... .......... .......... .......... ...T...... .......... ..P....... ..........
A_Kenya_042_2018             .......... .......... .......... .......... .......... .......... ...T...... .......... ..P....... ..........
A_Kenya_035_2018             .......... .........A .......... .......... .......... .......... ...T...... .......... .......... ..........
A_Kenya_036_2018             .......... .........A .......... .......... .......... .......... ...T...... .......... .......... ..........
A_Kenya_029_2018             .......... .......... .......... .......... .......... .......... ...T...... .......... .......... ..........
A_Kenya_025_2018             .......... .......... .......... .......... .......... .......... ...T...... .......... .......... ..........
A_Kenya_024_2018             .......... .......... .......... .......... .......... .......... ...T...... .......... .......... ..........
A_Kenya_020_2018             .......... .......... .......... .......... .......... .......... ...T...... .......... .......... ..........
A_Kenya_019_2018             .......... .......... .......... .......... .......... .......... ...T...... .......... .......... ..........
A_Kenya_017_2018             .......... .......... .......... .......... .......... .......... ...T...... .......... .......... ..........
A_Madagascar_883_2018        .......... .........A .......... .......... .......... .......... ...T...... .......... .......... ..........
A_South_Africa_5719_2018     .......... .......... .......... .......... .......... .......... ...T...... .......... ..P....... ..........
A_South_Africa_R07331_2018   .......... .........A .......... .......... .......... .....S.... ...T...... .......... .......... ..........
```

```
A_Uganda_6825_2018             .......... .........A .......... .......... .......... .......... ...T...... .......... .......... ..........
A_Bangkok_99_2018              .......... .......... .......... .......... .......... .......... ...T...... .......... .......... ..........
A_New_York_50_2018             .......... .......... .......... ......S... .......... .......... ...T...... ..I....... ..P....... ..........
A_Brazil_0382_2018             .......... .......... .....Y.... .......... .......... .......... ...T...... .......... ..P....... ..........
A_Sydney_1034_2018             .......... .........A .......... .......L.. .......... .......... ...T...... .......... ..P....... ..........
A_Pennsylvania_01_2018         .......... .......... .......... .......... .......... .......... ...T...... .......... .......... ..........
A_Florida_69_2018              .......... .........A .......... .......... .......... .......... ...T...... .......... .......... ..........
A_Uganda_6100_2018             .......... .........A .......... .......... .......... .......... ...T...... .......... .......... ..........
A_Egypt_458_2018               .......... .........A .......... .......... .......... .......... ...T...... .......... .......... ..........
A_Zhejiang-CHEN_2018           .......... .......... .......... .......... .......... .......... ...T...... .......... .......... ..........
A_Moscow_96_2018               .......... .........A .......... .......... .......... .......... ...T...... .......... .......... ..........
A_Brazil_2299_2018             .......... .........A .......... .......... .......... .......... ...T...... .......... .......... ..........
A_Indiana_21_2018              .......... .........A .......... .......... .......... .......... ...T...... .......... .......... ..........
A_Wyoming_04_2018              .......... .......... .......... .......... .......... .......... ...T...... .......... .......... ..........
A_Ohio_30_2018                 .......... .........A .......... ......S... E......... .......... .S.T...... .......... .......... ..........
A_New_Jersey_19_2018           .......... .......... .......... .......... .......... .......... ...T...... .......... ..P....... ..........
A_Washington_73_2018           .......... .........A .......... .......... .......... .......... ...T...... .......... ..P....... ..........

                                      210        220        230        240        250        260        270        280        290        300
                               ....|....| ....|....| ....|....| ....|....| ....|....| ....|....| ....|....| ....|....| ....|....| ....|....|
A_Michigan_45_2015_6B.1        VGTSRYSKKF KPEIATRPKV RDQEGRMNYY WTLVEPGDKI TFEATGNLVV PRYAFTMERN AGSGIIISDT PVHDCNTTCQ TPEGAINTSL PFQNIHPITI
A_Kenya_066_2018               .......... .......... .......... .......... .......... .......... T...V..... .......... .........P ....V.....
A_Kenya_047_2018               .......... .......... .......... .......... .......... .......... .......... ..Y....... .......... ....V.....
A_Kenya_059_2018               .......... .......... .......... .......... .......... .......... .......... ..Y....... .......... ....V.....
A_Kenya_045_2018               .......... .......... .......... .......... .......... .......... .......... ..Y....... .......... ....V.....
A_Kenya_046_2018               .......... .......... .......... .......... .......... .......... .......... ..Y....... .......... ....V.....
A_Kenya_039_2018               .......... .......... .......... .......... .......... .......... .......... ..Y....... .......... ....V.....
A_Kenya_041_2018               .......... .......... .......... .......... .......... .......... .......... ..Y....... .......... ....V.....
A_Kenya_027_2018               .......... .......... .......... .......... .......... .......... .......... .......... .......... ....V.....
A_Kenya_055_2018               .......... .......... .......... .......... .......... .......... .......... .......... .......... ....V.....
A_Kenya_060_2018               .......... .......... .......... .......... .......... A......... .......... .......... .S........ ....V.....
A_Kenya_056_2018               .......... .......... .......... .......... ....P..... .......... .......... .......... .......... ....V.....
A_Kenya_063_2018               .......... .......... .......... .......... .......... .......... .......... .......... .......... ....V.....
A_Kenya_028_2018               .......... .......... .......... .......... .......... .......... .......... .......... .......... ....V.....
A_Kenya_013_2018               .......... .......... .......... .......... .......... .......... .......... .......... .......... ....V.....
A_Kenya_065_2018               .......... .......... .......... .......... .......... .......... .......... .......... .......... ....V.....
A_Kenya_064_2018               .......... .......... .......... .......... .......... .......... .......... .......... .......... ....V.....
A_Kenya_062_2018               .......... .......... .......... .......... .......... .......... .......... .......... .......... ....V.....
A_Kenya_057_2018               .......... .......... .......... .......... .......... .......... .......... .......... .......... ....V.....
A_Kenya_051_2018               .......... .......... .......... .......... .......... .......... .......... .......... .......... ....V.....
A_Kenya_043_2018               .......... .......... .......... .......... .......... .......... .......... .......... .......... ....V.....
A_Kenya_042_2018               .......... .......... .......... ....K..... .......... .......... .......... .......... .......... ....V.....
A_Kenya_035_2018               .......... .....I.... .......... .......... .......... .......... .......... .......... .......... ....V.....
A_Kenya_036_2018               .......... .....I.... .......... .......... .......... .......... .......... .......... .......... ....V.....
A_Kenya_029_2018               .......... .......... .......... .......... .......... .......... .......... .......... .......... ....V.....
A_Kenya_025_2018               .......... .......... .......... .......... .......... .......... .......... .......... .......... ....V.....
A_Kenya_024_2018               .......... .......... .......... .......... .......... .......... .......... .......... .......... ....V.....
A_Kenya_020_2018               .......... .......... .......... .......... .......... .......... .......... .......... .......... ....V.....
A_Kenya_019_2018               .......... .......... .......... .......... .......... .......... .......... .......... .......... ....V.....
A_Kenya_017_2018               .......... .......... .......... .......... .......... .......... .......... .......... .......... ....V.....
A_Madagascar_883_2018          .......... .......... .......... .......... .......... .......... .......... .......... .......... ....V.....
A_South_Africa_5719_2018       .......... .......... .......... .......... .......... .......... .......... .......... .A........ ....V..V..
A_South_Africa_R07331_2018     .......... .......... .......... .......... .......... .......... .......... .......... .......... ....V.....
A_Uganda_6825_2018             .......... .......... .......... .......... .......... .......... .......... .......... .......... ....V.....
A_Bangkok_99_2018              .......... .......... .......... .......... .......... .......... .......... .......... .......... ....V.....
A_New_York_50_2018             .......... .......... .......... .......... .......... .......... .......... .......... .......... ....V.....
A_Brazil_0382_2018             .......... .......... .......... .......... .......... .......... .......... .......... .A........ ....V..V..
A_Sydney_1034_2018             .......... .......... .......... .......... .......... .......... .......... .......... .......... ....V.....
A_Pennsylvania_01_2018         .......... .......... .......... .......... .......... .......... .......... .......... .......... ....V.....
A_Florida_69_2018              .......... .......... .......... .......... .......... .......... .......... .......... .......... ....V.....
A_Uganda_6100_2018             .......... .......... .......... .......... .......... .......... .......... .......... .......... ....V.....
A_Egypt_458_2018               .......... .......... .......... .......... .......... .......... .......... .......... .......... ....V.....
A_Zhejiang-CHEN_2018           .......... .......... .......... .......... .......... .......... .......... .......... .......... ....V.....
A_Moscow_96_2018               .......... .......... .......... .......... .......... .......... .......... .......... .......... ....V.....
A_Brazil_2299_2018             .......... .......... .......... .......... .......... .......... .......... .......... .......... ....V.....
A_Indiana_21_2018              .......... .......... .......... .......... .......... .......... .......... .......... .......... ....V.....
A_Wyoming_04_2018              .......... .......... .......... .......... .......... .......... .......... .......... .......... ....V.....
A_Ohio_30_2018                 .......... .......... .......... .......... .......... .......... .......... ..Y....... .......... ....V.....
A_New_Jersey_19_2018           .......... .......... .......... .......... .......... .......... .......... .......... .......... ....V.....
A_Washington_73_2018           .......... .......... .......... .......... .......... .......... .......... .......... .......... ....V.....

                                      310        320
                               ....|....| ....|....| ....|..
A_Michigan_45_2015_6B.1        GKCPKYVKST KLRLATGLRN VPSIQSR
A_Kenya_066_2018               .......... ....V...N. ...S...
A_Kenya_047_2018               .......... .......... I......
A_Kenya_059_2018               .......... .......... .......
A_Kenya_045_2018               .......... .......... .......
A_Kenya_046_2018               .......... .......... .......
A_Kenya_039_2018               .......... .......... .......
A_Kenya_041_2018               .......... .......... .......
A_Kenya_027_2018               .......... .......... .......
A_Kenya_055_2018               .......... ....V..... .......
A_Kenya_060_2018               .......... ....V..... .......
A_Kenya_056_2018               .......... ....V..... .......
A_Kenya_063_2018               .......... ....V..... .......
A_Kenya_028_2018               .......... ....V..... .......
A_Kenya_013_2018               .......... ....V..... .......
A_Kenya_065_2018               .......... ....V..... .......
A_Kenya_064_2018               .......... ....V..... .......
A_Kenya_062_2018               .......... ....V..... .......
A_Kenya_057_2018               .......... ....V..... .......
A_Kenya_051_2018               .......... ....V..... .......
A_Kenya_043_2018               .......... .......... .......
A_Kenya_042_2018               .......... .......... .......
A_Kenya_035_2018               .......... .......... .......
A_Kenya_036_2018               .......... .......... .......
A_Kenya_029_2018               .......... .......... .......
A_Kenya_025_2018               .......... .......... .......
A_Kenya_024_2018               .......... .......... .......
A_Kenya_020_2018               .......... .......... .......
A_Kenya_019_2018               .......... .......... .......
A_Kenya_017_2018               .......... .......... .......
```

```
A_Madagascar_883_2018        .......... .......... .......
A_South_Africa_5719_2018     .......... .......... .......
A_South_Africa_R07331_2018   .......... .......... .......
A_Uganda_6825_2018           .......... .......... .......
A_Bangkok_99_2018            .......... .......... .......
A_New_York_50_2018           .......... .......... .......
A_Brazil_0382_2018           .......... .......... .......
A_Sydney_1034_2018           .......... .......... .......
A_Pennsylvania_01_2018       .......... .......... .......
A_Florida_69_2018            .......... .......... .......
A_Uganda_6100_2018           .......... .......... .......
A_Egypt_458_2018             .......... .......... .......
A_Zhejiang-CHEN_2018         .......... .......... .......
A_Moscow_96_2018             .......... .......... .......
A_Brazil_2299_2018           .......... .......... .......
A_Indiana_21_2018            .......... .......... .......
A_Wyoming_04_2018            .......... .......... .......
A_Ohio_30_2018               .......... .......... .......
A_New Jersey_19_2018         .......... .......... .......
A_Washington_73_2018         .......... .......... .......
```
